# One Size Doesn’t Fit All: Variability in Autistic Children’s Response to Pivotal Response Treatment

**DOI:** 10.3390/bs15121629

**Published:** 2025-11-27

**Authors:** Rachel K. Schuck, Ella Jevtić, Emily F. Ferguson, Maria Estefania Millan, Devon M. Slap, Mirko Uljarević, Jennifer M. Phillips, Antonio Y. Hardan, Grace W. Gengoux

**Affiliations:** Department of Psychiatry and Behavioral Sciences, Stanford University, Stanford, CA 94305, USA

**Keywords:** autism, pivotal response treatment, response to intervention, reliable change index, language

## Abstract

Pivotal Response Treatment (PRT) is a naturalistic developmental behavioral intervention designed to strengthen autistic children’s social communication skills. Few studies have examined which children benefit the most from PRT and which characteristics are associated with meaningful progress. We analyzed data from 23 children with autism and significant language delay who had been randomized to receive PRT in a previously completed 24-week randomized controlled trial of parent training and clinician-delivered intervention. Participants were categorized as intervention responders and non-responders based on the demonstration of meaningful improvement (or lack thereof) in social communication using the MacArthur-Bates Communicative Development Inventories (MCDI) Reliable Change index scores and clinician determination based on review of language samples and the Clinical Global Impressions—Improvement Scale (CGI). Baseline child characteristics associated with being a responder were assessed. Sixteen participants were responders on the language sample, ten on the MCDI, and sixteen on the CGI. Nine were consistent responders across all three measures; six were consistent non-responders. Verbal ability at baseline was associated with being a responder across all measures. In our small sample, baseline verbal ability was associated with being a responder to PRT, though categorization as a responder differed somewhat based on outcome measure. Future research should explore responder profiles specifically in children who are nonspeaking to inform the development of more effective supports for this group.

## 1. Introduction

Social communicative challenges are a hallmark of autism (American Psychiatric Association, 2013). Given that language and communication guide social interaction, a variety of interventions have been developed to target these areas, in the hopes that improvements will eventually lead to increased independence, enhanced quality of life, and overall better outcomes. Interventions to target language and social communication include speech and language therapy, developmental relationship interventions, and behavioral interventions. Several meta-analyses of group-design studies have demonstrated the effectiveness of behavioral interventions (Eckes et al., 2023; Makrygianni et al., 2018). Given the significant heterogeneity of autism with regard to phenotypic presentation and outcomes (Lombardo et al., 2019), including pronounced individual differences in intervention outcomes (Vivanti et al., 2014b), there has been increased focus on characterizing predictors of intervention response (Chetcuti et al., 2025). However, few studies have adopted an individual differences approach, which is essential for identifying subgroups of children who are likely to benefit most from specific interventions. This study aimed to identify and characterize responders—i.e., children who exhibited meaningful gains in the primary outcome (social communication abilities)—to an evidence-based naturalistic behavioral intervention, Pivotal Response Treatment (PRT; L. K. Koegel et al., 2016).

### 1.1. Behavioral Intervention

Behavioral interventions are a common approach to early intervention for children with autism (Monz et al., 2019). These interventions utilize principles of reinforcement to impact learning and behavior. Behavioral interventions can lead to benefits in areas such as expressive and receptive language, cognitive abilities, and adaptive skills (Eckes et al., 2023; Makrygianni et al., 2018). Early behavioral interventions tended to be clinician-directed and often utilized Discrete Trial Training (Smith, 2001), wherein skills are broken up into component parts that are practiced through repeated trials, usually using external reinforcers (Landa, 2018). Due to concerns that these traditional methods were resulting in low motivation (R. L. Koegel et al., 1998) and limited generalization of skills to new settings (Smith, 2001), a new class of interventions was developed that integrated ABA with principles of child development. These interventions—Naturalistic Developmental Behavioral Interventions (NDBIs; Schreibman et al., 2015)—emphasize following the child’s lead, practicing skills in natural environments, and providing naturalistic reinforcement, and have been shown to benefit autistic children (Crank et al., 2021; Tiede & Walton, 2019).

One common NDBI is Pivotal Response Treatment (PRT). PRT focuses on targeting pivotal areas to increase motivation and uses the following principles to guide implementation: acquiring the child’s attention and using clear prompts, following the child’s lead and gaining shared control of the activity, offering easy and hard tasks, and providing access to a direct, natural reinforcer for reasonable attempts (L. K. Koegel et al., 2016). Sessions are play-based, with an adult following PRT motivational techniques to create opportunities for language development. Parent training is an integral part of PRT (and all NDBIs), where parents learn the skills necessary to implement the intervention in interactions with their child outside of formal therapy sessions.

Analyses of PRT studies have consistently shown positive results in both clinician- and caregiver-reported measures, in addition to behavioral coding of child language. These results have been demonstrated in numerous single-subject design trials (see Bozkus-Genc & Yucesoy-Ozkan, 2016 for a review) and in more recent randomized controlled trials (RCTs; e.g., Hardan et al., 2015; Mohammadzaheri et al., 2014; van den Berk-Smeekens et al., 2021; Wang et al., 2024). Meta-analyses of PRT provide some evidence of its effectiveness in improving several areas of development, including language, communication, and social interaction (Ona et al., 2020; Uljarević et al., 2022). Furthermore, a variety of studies have demonstrated that many participating parents are able to learn to implement PRT with fidelity (e.g., Barrett et al., 2020; Gengoux et al., 2019; Hardan et al., 2015).

### 1.2. Predictors of Response to Behavioral Intervention

Autism is recognized as a vastly heterogeneous condition in regard to the onset, concurrent presentation, and trajectories of autistic features and co-occurring conditions (Chetcuti et al., 2025; Lombardo et al., 2019; Nordahl et al., 2022; Spackman et al., 2022). Given this heterogeneity, the notion that all autistic children will respond similarly to the same intervention is untenable. This realization has put the need to understand what works for whom and why into sharp focus (Vivanti et al., 2014b), with the hope of ultimately transitioning toward providing highly tailored, individualized supports. Thus, it is encouraging that a number of primary empirical studies and recent meta-analyses have started to explore predictors of meaningful intervention response. Two methods are generally used to assess predictors of response: (a) associations between baseline characteristics and outcomes at the group-level (e.g., Chetcuti et al., 2025), and (b) subgrouping participants into responders (those who demonstrate clinically/statistically meaningful improvement) and non-responders (those who do not demonstrate meaningful improvement) and assessing baseline predictors of belonging to these subgroups (e.g., Sherer & Schreibman, 2005). In both methods, response to intervention is defined using whatever the (primary) intended outcomes are, which vary across interventions as well as across clinicians and researchers.

### 1.3. Group-Level Predictors

Recent meta-analyses of behavioral interventions have emphasized some factors that influence outcomes post-intervention. In one study, researchers found that children with more advanced baseline language skills demonstrate significantly greater gains in behavioral outcomes than children with fewer language skills (Eckes et al., 2023). Another recent meta-analysis found that children who exhibited higher cognitive, language, adaptive functioning, motor skill, and developmental abilities also demonstrated more beneficial effects post-intervention (Chetcuti et al., 2025). Additionally, longer intervention duration and greater total hours of intervention were associated with improved outcomes across intervention types (Chetcuti et al., 2025).

Several studies have specifically looked at variables associated with improved outcomes in response to NDBIs. For example, in a trial of the Early Start Denver Model (ESDM), functional use of objects and imitation were related to gains in non-verbal skills, whereas fewer/less significant autistic features were predictive of gains in expressive language (Vivanti et al., 2013). On the other hand, in another ESDM study, Vivanti et al. (2014a) did not find evidence of baseline age or expressive and receptive language skills predicting changes in the primary outcome measures. In a study of JASPER, Panganiban and Kasari (2022) identified variables such as play diversity, requesting gestures, and fine motor skills as significant factors that can predict language gains in children who receive the intervention. When comparing group-delivered ESDM and Early Intensive Behavioral Intervention (EIBI), Bent et al. (2024) found that increased sustained attention at baseline was associated with non-verbal developmental quotient outcomes in the ESDM group, whereas social attention was linked to gains in verbal developmental quotient and adaptive behavior outcomes for both groups. Caregiver variables have also been investigated as potential predictors of response. For example, in a study of Project ImPACT, children of parents with lower baseline stress exhibited significantly greater social gains (Stadnick et al., 2015).

With regard to PRT, a recent meta-analysis of RCTs found that only four studies assessed outcome predictors at the group level (Uljarević et al., 2022). Findings were inconclusive; some studies found evidence that lower cognitive ability was related to greater improvements, and others reported the opposite or no relation. In an uncontrolled trial of PRT, Fossum et al. (2018) found that those with the greatest gains in expressive language skills had higher baseline expressive language, cognitive abilities, and toy contact, and lower levels of stereotyped vocalizations.

Overall, these research findings have identified a wide range of potential predictors associated with behavioral intervention outcomes for autistic children. While some studies suggest that factors such as baseline language skills, cognitive abilities, and caregiver stress influence these outcomes, others report inconsistent or contradictory results. This variability underscores the need for a more comprehensive understanding of these predictors—especially at the individual level—which could ultimately improve the efficacy of interventions.

### 1.4. Responder vs. Non-Responder Subgrouping

While the aforementioned studies provide important information about responsivity to behavioral intervention, group-level associations are limited in their ability to identify for whom an intervention will work (Chetcuti et al., 2025; Vivanti et al., 2014b). It has been argued that transitioning toward characterizing profiles of intervention responders—ultimately, precision medicine in autism research and intervention—is essential (Beversdorf & Missouri Autism Summit Consortium, 2016; Loth et al., 2016). One approach to characterizing responders is via distribution-based methods, such as deriving the Reliable Change Index (RCI; Jacobson & Truax, 1991). These methods incorporate the standard error of the measurement (e.g., test–retest reliability) to estimate whether the difference between two scores is likely to have been due to chance by assessing whether it falls within or outside a specified confidence interval (Frazier et al., 2025a). Alternatively, in contexts where criteria for clinically meaningful differences are available, for example, clinical judgment, anchor-based methods have been utilized to identify intervention responders (Chatham et al., 2018).

A limited number of studies have looked at predictors of being a responder versus a non-responder to NDBI. An anchor-based approach was used by Asta et al. (2024) to categorize ESDM recipients into three categories of response. “Strong” responders were defined as those who no longer met diagnostic criteria for autism^1^ and had substantial improvements in language, “moderate” responders were those who decreased in diagnostic autism severity and showed some improvement in language or non-verbal communication, and “poor” responders were those who still fully met autism diagnostic criteria and had acquired little verbal or non-verbal communication. Factors associated with better response included baseline cognitive ability, expressive and receptive language, and fewer repetitive and restricted behaviors. Several studies have taken a similar approach to PRT, though the evidence base is still quite limited (Lei & Ventola, 2017), with most focusing on the profile identified by Sherer and Schreibman (2005). In this early investigation, the authors analyzed 28 participants from prior PRT studies and identified the six children who made the greatest increase in language scores as “exceptional responders” and the five who made little or no gains as “poor responders.” Videotaped structured lab observations were used to code the frequency of several relevant child behaviors, including toy contact, approach and avoidance, and self-stimulatory behaviors. At baseline, responders had higher interest in toys, less avoidance of others, and greater rates of verbal self-stimulatory behavior compared to non-responders. The authors then prospectively recruited autistic children matching these profiles into a new study. In this follow-up, the children who matched the baseline characteristics of the responder group demonstrated improvements across various outcomes, whereas the non-responders did not. Schreibman et al. (2009) further investigated these profiles by recruiting children who matched the non-responder profile except in one area (either toy contact or avoidance). Amongst the children matching an otherwise non-responder profile, those with high toy contact behavior had more favorable outcomes, while those who matched the non-responder profile except for having low avoidance of adults did not (Schreibman et al., 2009). While these studies provide useful preliminary information regarding responder profiles, all utilize only anchor-based methods of categorizing responders, and it is unclear how they might align with distribution-based methods, such as RCI. Additionally, the profile identified by Sherer and Schreibman (2005) relied solely on metrics derived from behavioral coding from videos; such labor-intensive coding is unlikely to be feasible for community providers when making intervention recommendations.

### 1.5. Current Study

This secondary analysis aims to address some of the identified limitations of previous studies by examining responders versus non-responders to PRT in a previously completed RCT (Gengoux et al., 2019). The initial study found that children in the PRT group significantly improved in their social communication skills compared to the control group across multiple outcome measures (Gengoux et al., 2019). In the current study, we aimed to (a) identify responders using both distribution-based and anchor-based methods, and (b) explore baseline predictors of response. An improved understanding of these predictors may identify gaps in PRT research and facilitate the development of more personalized intervention recommendations, leading to better outcomes for children participating in early intervention programs.

## 2. Materials and Methods

All parents provided informed consent before participating in the study. The study was approved by the Stanford University Institutional Review Board.

### 2.1. Participants

This study contains data from participants from a previously completed RCT of PRT parent training and clinician-delivered intervention (Gengoux et al., 2019). Participants included in this report were those who were randomized to the intervention group and completed the 24-week study (n = 23). An existing community diagnosis of autism was required to participate; autism traits were confirmed using the Autism Diagnostic Observation Schedule (ADOS; Lord et al., 2012) and Autism Diagnostic Interview–Revised (ADI-R; Lord et al., 1994). Participants also needed to exhibit significant language delay, as evidenced by scores on the Preschool Language Scale (Zimmerman et al., 2011). The majority of the child participants were male (n = 21; 91.3%), and most participating parents were female (n = 15, 65.2%). Most children were Asian (n = 12, 52.2%), roughly a quarter were White (n = 6, 26.1%), two were Hispanic (8.7%), and the remaining three were biracial, Native Hawaiian/Pacific Islander, and “other” race. Participants ranged from 30 to 71 months at baseline (M = 49.52, SD = 11.18). The full-scale developmental quotient (FSDQ; calculated from Mullen Scales of Early Learning (MSEL) scores) indicated that all children exhibited developmental delays (FSDQ Range: 20.45–64.29, M = 44.72, SD = 11.82).

### 2.2. Procedures

All parents completed a questionnaire battery at baseline, halfway through the study (week 12), and after study completion (week 24). Several child assessments were also administered at these timepoints. Participants did not receive any compensation for participating in the study.

The study intervention consisted of a package of PRT parent training and in-home, clinician-delivered PRT targeting spoken language (Gengoux et al., 2019). The intervention included 12 weeks of intensive PRT (10 h in-home + weekly parent training), followed by 12 weeks of reduced intensity (5 h in-home + monthly parent training). All PRT intervention was focused on improving spoken language and followed the six principles of PRT (providing clear learning opportunities, allowing for child choice/following the child’s lead, varying between easy and difficult tasks, contingent reinforcement, provision of natural reinforcers, and reinforcing attempts; L. K. Koegel et al., 2016).

### 2.3. Measures

#### 2.3.1. Manually Coded Utterances from Structured Lab Observation (SLO)

During the SLO, parents and children are video recorded for ten minutes with a standardized set of toys. Parents are instructed to play with their child using the toys provided and to encourage their child to talk/communicate as much as possible during this playtime. Utterances made during SLOs were manually coded as unintelligible, imitative, verbally prompted, non-verbally prompted, and spontaneous. Coding was conducted by two blind coders. Approximately 30% of videos were independently double-coded; agreement ranged from acceptable to excellent (Gengoux et al., 2019).

#### 2.3.2. MacArthur Bates Communicative Development Inventories—Words and Sentences (MCDI; Fenson et al., 2007)

This parent-report questionnaire includes a checklist of 680 words (where the parent can indicate whether the child can say each word) and a section that evaluates grammar and sentence structure, including word combinations and complexity. Only the vocabulary checklist was used in the current study.

#### 2.3.3. Clinical Global Impressions (CGI; Guy, 1976)

The CGI assesses participants for: Severity of Illness and Global Improvement, scored on a seven point scale (1: Normal, Not at all ill/Very much improved; 2: Borderline mentally ill/Much improved; 3: Mildly ill/Minimally improved; 4: Moderately ill/No change; 5: Markedly ill/Minimally worse; 6: Severely ill/Much worse; 7: Among the most extremely ill subjects/Very much worse). The current study focused on the CGI Global Improvement subscale (CGI) related to communication. To complete the CGI, a board-certified child psychiatrist naïve to group assignment met with caregivers and children for a brief (~5 min) interview/observation.

#### 2.3.4. Mullen Scales of Early Learning (MSEL; Mullen, 1995)

The MSEL is a clinician-administered developmental assessment measuring the following five domains: gross motor, visual reception, fine motor, receptive language, and expressive language. Each subscale includes items corresponding to different ages and developmental milestones, intended for administration to children from birth to 68 months of age. To calculate the developmental quotient used in the study, the raw scores are converted into age equivalents, which were divided by the participant’s chronological age and multiplied by 100.

#### 2.3.5. Social Responsiveness Scale, 2nd Edition, Preschool Version (SRS-2; Constantino, 2012)

The SRS-2 is a 65-item parent-report questionnaire designed to assess autistic traits (i.e., the presence of social communication difficulties and restricted and repetitive behaviors). Raw scores are then converted into T-scores, which were used in the current study.

#### 2.3.6. Parent PRT Fidelity During SLO

Parents’ fidelity of implementation of PRT during SLOs was scored by trained raters naïve to group assignment and timepoint. These coders were different than the utterance coders. Moderate agreement (as defined by McHugh, 2012) was obtained (see Gengoux et al., 2019 for more details). Parents’ performance was scored across six motivational PRT principles: clear prompting, providing maintenance and acquisition tasks, child choice, contingency, natural reinforcement, and reinforcing attempts. Almost all parents met PRT fidelity at week 24 (21/23; Gengoux et al., 2019).

### 2.4. Data Analysis

#### 2.4.1. Defining Responders to PRT

We categorized “responders” in three ways: (1) using Reliable Change (RC) scores based on pre- and post-MCDI-WS scores, (2) clinician rating of communication being “very much improved” or “much improved” on the CGI, and (3) qualitative categorization of utterance response patterns during SLOs. Week 24 data was used as the “post” timepoint across all three outcomes.

##### MCDI Responders

MCDI-WS RC z-scores were calculated using the formula put forth by Jacobson and Truax (1991), where RC is equal to the post-pre score difference divided by the standard error of difference between the two test scores (which incorporates both the standard deviation of the measure at baseline as well as the established test–retest reliability of the instrument, which is reported to be 0.95 (Fenson et al., 2007)). Participants were categorized as responders if their RC z-score was greater than 1.96 (the critical value for Z-scores at the 95% confidence interval, which suggests that the difference in scores represents a real change that is unlikely to be due to chance at the *p* < 0.05 level; Jacobson & Truax, 1991).

##### CGI Responders

If the blinded clinician rated a child’s communication skills as very much or much improved at week 24, the child was considered a responder; any other score was considered a non-responder.

##### SLO Responders

For determining responsivity based on the SLO, we opted not to use simple post-pre difference scores as indicators of meaningful change, as specific patterns of utterance changes could theoretically imply different patterns of improvement (or lack thereof). For example, a child who has a large decrease in imitative utterances but a moderate increase in both verbally prompted and spontaneous language would likely be perceived as making an improvement, even if the overall number of utterances was slightly decreased. However, if a child demonstrates the same decrease in imitative language while also increasing in unintelligible utterances, that would likely be seen as a lack of meaningful improvement.

Thus, we decided to have expert clinicians naïve to group assignment review changes in patterns of utterances pre- and post-intervention. Two clinicians with extensive PRT experience (both Master’s level, one a BCBA) reviewed bar graphs of utterances at baseline and week 24 for all randomized participants (24 in the treatment group and 24 in the control group). Each bar was labeled with the frequency of each utterance type so figures could be easily compared between timepoints; however, graphs did not contain any identifying information. The clinicians were asked to categorize children as “verbal responder” (i.e., the child appeared to improve in intelligible utterances), “unintelligible responder” (i.e., the child improved in number of unintelligible but not intelligible utterances—likely signifying the child acquired intent to communicate vocally), and “non-/limited responder” (i.e., the child did not appear to make intelligible or unintelligible gains). Clinicians were instructed to use their expert judgment in making these categorizations and were not provided with any specific instructions regarding what counted as “meaningful” change. The two clinicians disagreed on three children’s classifications; in these cases, the first author (RKS) reviewed the graphs and determined the final classification. The three participants who were determined to be “unintelligible responders” on the SLO were classified as non-responders based on both the MCDI and CGI (see Table 1); therefore, for predictor analyses (described below), these participants were considered non-responders.

#### 2.4.2. Statistical Analysis

The number of participants considered responders based on each outcome variable is reported. A set of independent sample t-tests was run to assess whether baseline variables were associated with being a responder on the MCDI, CGI, or SLO. Relevant baseline variables included child age, developmental level (i.e., MSEL verbal and non-verbal developmental quotient), autistic features (SRS-2 repetitive and restrictive behavior subscale T-score and social communication index T-Score), and baseline intelligible utterances made during SLO. The total number of weekly ABA hours, as well as the week 24 parent PRT fidelity, were also assessed, since these could impact the amount and quality of intervention children were receiving. Where Levene’s Test for Equality of Variances was significant, equal variances were not assumed. Child gender was not analyzed because of the small number of female participants (n = 2).

## 3. Results

### 3.1. Number of Responders

Sixteen out of twenty-three (69.57%) participants were responders based on the SLO classifications (see Table 1). Ten out of twenty-three (43.48%) were categorized as responders based on the RC calculations from the MCDI-WS vocabulary scores. Sixteen out of twenty-two (72.73%; one participant did not complete the week 24 CGI) were categorized as responders based on the CGI-Communication classification.

### 3.2. Consistency of Responder Categorization Across Measures

Fifteen participants (65.22%) had consistent responder/non-responder categorizations across all three communication outcome variables. Nine of these fifteen participants were categorized as responders across all outcomes; the other six were consistent non-responders. Six of the eight participants with inconsistent categorizations were non-responders based on the MCDI but responders on the SLO and CGI. The participant who did not complete the week 24 CGI also showed a similar pattern of being a non-responder on the MCDI but a responder on the SLO. The last child (#12 in Table 1) showed a unique pattern of being a responder on the MCDI and CGI but not the SLO.

### 3.3. Predictors

Verbal DQ at baseline was a significant predictor of being a responder on the SLO (*t*(21) = 2.57, *p* = 0.02; SLO responders: M = 43.63, SD = 12.45; SLO non-responders: M = 26.46, SD = 19.29). Two baseline variables predicted being a responder on the MCDI-WS: MSEL Verbal DQ (*t*(21) = 2.42, *p* = 0.03) and number of intelligible utterances made during the baseline SLO (*t*(21) = 2.75, *p* = 0.01); see Table 2. The same two variables were also significant predictors of being a responder on the CGI (Verbal DQ: *t*(20) = 5.21, *p* < 0.001; intelligible utterances: *t*(19.46) = 4.58, *p* < 0.001). Across both outcome measures, participants who were considered responders had higher baseline Verbal DQ (MCDI-WS responders: M = 46.99, SD = 13.16; MCDI-WS non-responders: M = 31.81, SD = 16.16; CGI responders: M = 46.73, SD = 11.53; CGI non-responders: M = 19.76, SD = 8.34) and more intelligible utterances at baseline (MCDI-WS responders: M = 23.20, SD = 15.43; MCDI-WS non-responders: M = 9.08, SD = 9.11; CGI responders: M = 20.50, SD = 13.47; CGI non-responders: M = 3.50, SD = 3.83). 

Participant data for relevant outcome and predictor variables can be found in the Supplemental Materials.

## 4. Discussion

This secondary analysis aimed to identify responders to a 24-week parent training and clinician-delivered package of PRT (Gengoux et al., 2019). The original study found significant improvements in socio-communication in the PRT group compared to the control group, but did not assess profiles of improvement at the individual level. In the current study, we found that between 43 and 73% of participants were categorized as Responders depending on the outcome measure used to classify participants. We also found that higher baseline verbal ability was a significant predictor of being classified as a responder to PRT. These results highlight that accounting for individual differences in verbal skills may be important when designing and implementing interventions and contribute to our understanding of response profiles. Additionally, our findings have the potential to facilitate more effective early intervention matching and promote personalized intervention recommendations to ensure better participant outcomes.

The majority of participants (65.22%) showed consistent categorizations across the MCDI, CGI, and SLO. Importantly, 39.13% of participants were consistently identified as responders, whereas 26.09% were consistently identified as non-responders. This highlights the utility of considering profiles of responsivity as opposed to defining responsivity based on only one outcome measure, particularly given that traditional norming processes used for common behavioral intervention outcome measures are limited (for example, by accounting for only sex or age or by overreliance on simplistic linear modeling; Frazier et al., 2025b). Nevertheless, it is still critical to consider each individual outcome measure and to explore discrepancies. The three outcome measures used to characterize responders are discussed below.

### 4.1. Comparing Responders According to Outcome Measure

Based on the language sample captured during the SLO—an anchor-based metric of responsivity—the majority of participants were categorized as responders. Given that parents were instructed to encourage their child to communicate as much as possible (with most meeting fidelity of PRT implementation during this 10 min segment; Gengoux et al., 2019), the SLO appears to capture whether the child learns the behavioral contingency (i.e., if you communicate, you receive the natural reinforcer) in PRT sessions. This is especially true if one considers that the three Unintelligible Responders (those who increased in unintelligible utterances but not intelligible words) likely also learned this contingency. However, it is possible that the SLO is too highly context-bound (i.e., it closely mimics the intervention setting) to be an ideal metric of responsivity. Context-bound outcome measures have a higher likelihood of showing larger effect sizes (Crank et al., 2021; Sandbank et al., 2023), but such findings may not generalize to less context-bound settings (Ferguson et al., 2025). If we are interested in assessing responsivity in terms of functional outcomes in children’s everyday lives, it is important to augment the SLO with natural language samples collected in home, school, and community settings to capture generalizability in daily life.

Based on parent-reported words produced on the MCDI, 43.48% of participants were categorized as responders. Crucially, this is the only distribution-based method of categorizing responders in the current study. MCDI responsivity was determined by calculating Reliable Change z-scores, which take into account the baseline variation in scores as well as the test–retest reliability of the instrument. The MCDI-WS has quite a high test–retest reliability (0.95; Fenson et al., 2007), so we have limited concerns about measurement error in our RCI scores. However, some researchers warn that RCI can underestimate treatment effects (Blampied, 2022; McAleavey, 2024). Indeed, most of the participants with inconsistent classifications were found to be non-responders on the MCDI yet responders on the CGI and SLO.

Our last metric of responsivity, the CGI-I, was rated by an expert clinician naïve to group assignment after a brief interview with the caregiver and child observation. Sixteen participants were classified as responders on the CGI, meaning they were rated as being “very much” or “much” improved in communication. In contrast to the context-boundedness of the SLO, the CGI provides a more holistic measure of communication that is likely more representative of everyday life. Nevertheless, almost all of the CGI responders were also responders on the SLO, suggesting these two measures are highly aligned (at least in our small sample). This suggests more research is needed to determine the degree to which these outcome measures are truly related to outcomes in daily life versus bound to the intervention context. Interestingly, all 10 MCDI Responders were also classified as CGI Responders, which suggests that the CGI is sensitive to large gains in vocabulary. However, the CGI may be detecting nuanced improvements in communication that the MCDI might miss, given its focus on the number of words produced, such as increased engagement in social communication and more reliable language, even if children’s vocabulary has not meaningfully increased.

These findings highlight the importance of carefully operationalizing and measuring the concept of an intervention “responder.” For instance, in the context of PRT, is it more important that children are able to understand an intervention’s behavioral contingency, that their vocabulary substantially increases, or that their communication in daily life improves? The relevance of each of these outcomes may vary based on time and by child, and it may be that it is the constellation of these factors that is more important than any single outcome. Additionally, some of the most frequently used outcome measures in adaptive social communication intervention research (e.g., the Vineland Adaptive Behavior Scales-Third Edition (Vineland-3; Sparrow et al., 2016) and the Adaptive Behavior Assessment System-Third Edition (ABAS-3; Harrison & Oakland, 2015)) may lack utility in that they do not provide enough detail about specific subdomains of social communication (Frazier et al., 2025c). Lastly, even if we are able to identify valid and reliable outcome measures, it is important to ensure that they are truly meaningful and align with the autistic community’s values (Baiden et al., 2025; Nicolaidis et al., 2025; Sulek et al., 2025). For example, other variables that are less often assessed within the context of behavioral intervention trials may be highly relevant to take into consideration, such as child and family quality of life.

### 4.2. Predictors of Responsivity

Even though responder classification varied somewhat based on outcome measure, it is clear that there are some children for whom the PRT package did not lead to significant improvements. The likelihood of being a responder was not related to parent PRT fidelity at week 24 or the number of hours of outside ABA. Instead, our preliminary investigation into correlates of PRT responsivity pointed to the importance of baseline verbal communication skills. Several other baseline characteristics, including social challenges as measured by the SRS-2 and non-verbal developmental quotient on the MSEL, approached significance, suggesting these factors should be further explored in future studies. 

Baseline verbal skills being a predictor of response aligns with existing research on behavioral interventions at the group level (Chetcuti et al., 2025; Eckes et al., 2023; Mandelli et al., 2025). This underscores the importance of considering verbal skills when making intervention recommendations and setting intervention goals and expectations. While it may be less likely for children with limited language skills to make significant progress in spoken language after any intervention, our findings documenting greater improvement in verbal individuals should be viewed as preliminary and require replication. Our results should certainly not be taken to imply that children with lower verbal ability will not at all benefit from PRT (or other NDBIs or behavioral interventions). Indeed, non-speaking children may need supports tailored specifically for minimally verbal individuals in order to benefit most. For instance, it is possible that longer duration or more intensive PRT would have resulted in improved response in the non-responders. Additionally, this finding is probabilistic, which means some children with very limited language abilities may indeed meaningfully improve in their spoken language skills if provided PRT. In any event, even if significant gains in spoken language may be less likely for children with more limited language skills, children of all language levels and their families may benefit from PRT in other ways, such as improvements in receptive language (Wang et al., 2024) or reductions in parental stress (Minjarez et al., 2013), though more research is needed regarding collateral gains related to PRT (Verschuur et al., 2014). Furthermore, while the PRT in our study was specifically targeting spoken language, PRT and other NDBIs can be used to address other essential domains, including peer relationships (Gengoux et al., 2021), parent responsivity (Shire et al., 2016), and academic engagement (Poyser, 2021).

Our findings, in conjunction with prior research on responsivity to behavioral intervention (Chetcuti et al., 2025; Eckes et al., 2023; Mandelli et al., 2025), reveal a gap in evidence-based practices for children with more profound language delays, a finding echoed in L. K. Koegel et al.’s (2019) systematic review. Given that interventions appear to confer fewer gains to children with lower verbal ability, significantly more research must be conducted with non-speaking/minimally speaking autistic children (as well as autistic adolescents and adults; Ferguson et al., 2024). With regard to future PRT and NDBI trials, researchers should consider collaborating with speech–language pathologists to incorporate robust Augmentative and Alternative Communication (AAC) systems into their study designs, which would align with calls from the autistic community (Schuck et al., 2024) to position AAC as an equally valid means of communication on par with spoken language (Donaldson et al., 2021). Indeed, emerging studies of NDBI plus AAC show promising outcomes (see Pope et al., 2025 for a review). Future studies could, for example, utilize Sequential Multiple Assignment Randomized Trials (SMART; Almirall et al., 2014) where an AAC device is introduced if a child appears to not be responding to spoken language-focused NDBI after a set period of time (e.g., Kasari et al., 2014). The identification of responders should include both anchor-based metrics (such as clinician and parent impressions) and distribution-based metrics (e.g., RCI on standardized measures) across a constellation of outcomes. 

It is also possible that young children with more limited communication skills would benefit from initial exposure to other kinds of interventions before participating in NDBI. For example, trials could first start with a period of more structured teaching to solidify understanding of behavioral contingencies or, alternatively, a non-behavioral intervention, such as DIR/FloorTime (Pajareya & Nopmaneejumruslers, 2011), where relationship-building and child–parent responsiveness are emphasized. Developing new interventions/supports to effectively address the needs of this population—particularly those that are informed by lived experience—is also of critical importance (Hajizadeh Saffar et al., 2025; Krishnamurthy et al., 2022).

### 4.3. Limitations

While this study presents very preliminary evidence of who might respond best to this type of PRT implementation, there are also several limitations to consider. First, the small sample size restricted the robustness of the analysis. A larger sample size is essential to replicate and further refine the profiles of responders and non-responders. It will also be important for future research to ensure the outcomes used to determine intervention responsivity are aligned with the priorities of the autistic/autism community (e.g., Baiden et al., 2025; Sulek et al., 2025). Additionally, RCI is generally seen as a robust way to estimate significant change accounting for measurement error (Frazier et al., 2025a). In the current study, RCI could only be calculated for one of our outcomes. While RCI does not supplant expert clinical judgment, it would be helpful if more studies in the future use measures where both traditional and regression-based RCI (Frazier et al., 2025a) can be calculated (i.e., measures with established test–retest reliability). Given that the study design included only children who received PRT, it is not possible to determine whether the response patterns observed are treatment-specific or if the children classified as non-responders would have demonstrated minimal response to other intervention approaches as well. For this reason, it will be important for future studies to include comparisons across treatment types. Additionally, in our study, all participants received less intensive PRT after the initial 12-week period; we therefore do not know how the non-responders would have responded if they had continued with the more intensive phase or whether greater intensity of PRT from the start would have led to different findings. Adaptive research designs could help address this question in the future. Lastly, while all participants in the study had significant language delay at baseline, if larger numbers of specifically non-speaking/minimally speaking autistic children are recruited in future studies, predictors of responsivity in this understudied group (Russell et al., 2019; Tager-Flusberg & Kasari, 2013) could be investigated to inform the development of more effective interventions tailored to their needs.

## 5. Conclusions

In this secondary analysis of a 24-week RCT evaluating a parent training and clinician-delivered PRT intervention (Gengoux et al., 2019), autistic children were categorized as responders based on three outcome measures (RC scores based on changes in the MCDI-WS vocabulary list, SLO utterance response patterns, and ratings of communication improvement on the CGI by a blinded clinician). About a quarter of the participants were consistently classified as “non-responders” across all three communication measures. Our findings revealed that higher verbal ability at baseline was a significant predictor of positive response to PRT. This study adds to our understanding of which children may benefit most from this type of behavioral intervention, and understanding the response profiles can promote more accurate assessments and personalized intervention recommendations. Future research should further explore responder profiles in this population, focusing on the development of treatment modifications to more directly address the needs of minimally verbal individuals.

## Figures and Tables

**Table 1:** Responder Categorization Based on Communication Outcome Measure.

Participant	SLO	MCDI Words and Sentences	CGI: Communication	Consistent Responder Across Measures	Consistent Non-Responder Across Measures
1	Yes	Yes	Yes	✓+	
2	Yes	Yes	Yes	✓+	
3	Yes	No	Yes		
4	Yes	No	Yes		
5	No	No	No		✓−
6	Yes	Yes	Yes	✓+	
7	Yes	Yes	Yes	✓+	
8	Yes	Yes	Yes	✓+	
9	No (UR *)	No	No		✓−
10	No (UR *)	No	No		✓−
11	Yes	Yes	Yes	✓+	
12	No	Yes	Yes		
13	Yes	No	Yes		
14	Yes	No	-		
15	No	No	No		✓−
16	Yes	No	Yes		
17	Yes	Yes	Yes	✓+	
18	Yes	No	Yes		
19	Yes	No	Yes		
20	No	No	No		✓−
21	No (UR *)	No	No		✓−
22	Yes	Yes	Yes	✓+	
23	Yes	Yes	Yes	✓+	
Total	16 (69.57%)	10 (43.48%)	16 (72.73%) ^ɑ^	9 (3.913%) ^ɑ^	6 (26.09%) ^ɑ^

Note. One participant did not complete the week 24 CGI. * Participants classified as “Unintelligible-Responders” on the SLO (n = 3) are counted as non-responders in this table and identified by “UR”. ✓+ corresponds to consistently being categorized as a responder across all three outcome measures; ✓− corresponds to consistently being categorized as a non-/limited responder. ^ɑ^ Because participant #14 did not complete the week 24 CGI, the percentage for CGI responders is out of 22 participants.

**Table 2:** Predictors of Response to PRT Across Communication Outcome Measures.

	SLO			
	*t* (df)	*p*	Mean Diff	Std. Error Difference
Age at Baseline	−0.74 (21)	0.47	−3.77	5.12
Nonverbal DQ	1.05 (7.97)	0.33	5.79	5.53
**Verbal DQ**	**2.57 (21)**	0.02 *	**17.17**	**6.68**
BL Intelligible Utterances on SLO	1.24 (21)	0.23	7.71	6.24
Baseline SRS-2 RRB T-Score	0.16 (21)	0.87	0.88	5.42
Baseline SRS-2 SCI T-Score	−1.07 (21)	0.30	−4.54	4.22
Hours of Weekly ABA	−0.59 (21)	0.56	−1.43	2.41
Parent PRT Fidelity at Week 24	1.54 (6.39)	0.17	6.96	4.54
	**MCDI-WS**			
	***t* (df)**	***p***	**Mean Diff**	**Std. Error Difference**
Age at Baseline	−0.16 (21)	0.88	−0.75	4.81
Nonverbal DQ	1.43 (21)	0.17	5.96	4.18
**Verbal DQ**	**2.42 (21)**	0.03 *	**15.18**	**6.29**
**BL Intelligible Utterances on SLO**	**2.75 (21)**	0.01 *	**14.12**	**5.14**
Baseline SRS-2 RRB T-Score	−0.79 (21)	0.44	−3.91	4.96
Baseline SRS-2 SCI T-Score	−1.86 (21)	0.08	−6.95	3.73
Hours of Weekly ABA	−1.66 (21)	0.11	−3.51	2.12
Parent PRT Fidelity at Week 24	1.19 (14.49)	0.26	3.31	2.79
	**CGI-Communication**			
	***t* (df)**	***p***	**Mean Diff**	**Std. Error Difference**
Age at Baseline	−1.00 (20)	0.33	−5.40	5.40
Nonverbal DQ	1.87 (20)	0.08	8.79	4.70
**Verbal DQ**	**5.21 (20)**	<0.001 *	**26.97**	**5.18**
**BL Intelligible Utterances on SLO**	**4.58 (19.46)**	<0.001 *	**17.00**	**3.71**
Baseline SRS-2 RRB T-Score	−0.32 (20)	0.76	−1.83	5.78
Baseline SRS-2 SCI T-Score	−1.55 (20)	0.14	−6.85	4.44
Hours of Weekly ABA	−0.28 (20)	0.78	−0.68	2.43
Parent PRT Fidelity at Week 24	1.48 (5.24)	0.20	7.71	5.22

Note. * *p* < 0.05. Bolded text in the table is used to highlight the significant variables. MCDI-WS = MacArthur Bates Communicative Development Inventories—Words and Sentences; DQ = developmental quotient as calculated from the Mullen Scales of Early Learning; SRS-2 = Social Responsiveness Scale, 2nd Edition; RRB = restricted and repetitive behavior; SCI = social communication index; ABA = applied behavior analysis intervention; PRT = Pivotal Response Treatment. MCDI and SLO analyses include 23 participants; only 22 participants had CGI data. In the analyses presented here, SLO “Responders” refers to participants who made meaningful gains in intelligible speech; Unintelligible Responders were categorized as non-responders.

## Data Availability

Data related to this study will not be made publicly available. Please contact the corresponding author with any inquiries related to data sharing.

## Note

1 We do not recommend using diagnostic criteria as an indicator of intervention response, as this implies that to be “less autistic” is an optimal outcome and could lead to providers encouraging their clients to mask autistic features.

## Supplementary Materials

The following supporting information can be downloaded at: https://www.mdpi.com/article/10.3390/bs15121629/s1.

## Abbreviations

The following abbreviations are used in this manuscript:

PRT	Pivotal Response Treatment
MCDI	MacArthur-Bates Communicative Development Inventories
CGI	Clinical Global Impressions
RCT	Randomized Controlled Trial
NDBIs	Naturalistic Developmental Behavioral Interventions
ABA	Applied Behavior Analysis
ESDM	Early Start Denver Model
EIBI	Early Intensive Behavioral Intervention
SLO	Structured Lab Observation
MSEL	Mullen Scales of Early Learning
SRS-2	Social Responsiveness Scale, 2nd Edition
RRB	Restricted and Repetitive Behavior
SCI	Social Communication Index
FSDQ	Full-Scale Developmental Quotient
RCI	Reliable Change Index
