# Supplementary material for: One Size Doesn’t Fit All: Variability in Autistic Children’s Response to Pivotal Response Treatment
Source: Behav Sci (Basel). 2025 Nov 27;15(12):1629. doi: 10.3390/bs15121629 (PMC12729598; doi:10.3390/bs15121629)
Supplement: Supplementary file 1 [file behavsci-15-01629-s001.zip › behavsci-3936239-supplementary.pdf]

Supplemental Table S1.
Participant Scores on Relevant Outcome Measures

	MCDI Words & Gestures				MCDI Words & Sentences		Utterances During Structured Lab Observation										CGI-I
Child	BL Words Understood	BL Words Produced	Wk24 Words Understood	Wk24 Words Produced	BL Words Produced	Wk24 Words Produced	BL Unintelligible	BL Modeled	BL VP	BL NVP	BL Spon.	Wk24 Unintelligible	Wk24 Modeled	WK24 VP	Wk24 NVP	Wk24 Spon.	Wk24
1	142	24	239	207	45	238	36	20	7	0	0	34	30	2	2	1	VM
2	226	97	245	217	91	273	38	4	4	0	0	35	27	9	4	0	VM
3	143	62	163	110	76	146	60	12	1	0	0	39	35	25	4	0	VM
4	199	121	256	190	185	235	47	18	9	0	1	15	24	17	9	8	M
5	108	8	94	29	16	35	57	5	2	0	0	44	4	0	0	0	Min
6	304	27	395	365	33	553	11	1	0	0	0	36	8	1	0	0	M
7	327	254	369	306	273	388	10	8	20	2	0	3	25	17	10	0	M
8	322	288	379	367	286	587	6	2	4	0	0	14	30	75	8	2	M
9	59	0	51	1	1	1	5	0	0	0	0	21	0	0	0	0	NC
10	26	0	63	1	0	5	48	0	0	0	0	59	1	0	0	0	Min
11	266	178	318	302	241	344	37	10	7	0	2	30	26	13	6	2	M
12	313	309	349	326	267	524	12	19	24	0	5	19	11	26	2	4	M
13	250	206	396	248	277	253	1	11	14	0	1	7	17	18	7	3	M
14	77	21	101	60	37	63	17	1	0	0	0	18	28	8	8	6	*
15	24	7	48	43	12	25	21	8	1	0	0	25	6	0	2	0	Min
16	354	143	379	305	225	285	43	8	1	0	0	42	26	5	0	0	M
17	246	88	286	222	105	238	39	13	6	0	0	46	37	8	1	1	M
18	193	91	308	164	118	194	34	7	5	0	0	59	24	3	0	0	M
19	330	279	370	356	471	498	18	3	5	0	0	34	11	4	1	2	M
20	33	6	53	6	8	7	140	1	0	0	0	100	0	0	0	0	Min
21	198	4	139	16	4	10	26	4	0	0	0	51	5	0	0	0	Min
22	371	293	321	312	230	522	26	12	22	3	4	12	50	45	15	10	VM
23	262	213	330	329	262	477	67	19	14	0	0	19	21	26	7	5	M

Note. MCDI=MacArthur-Bates Communicative Development Inventory; CGI-I=Clinical Global Impressions—Improvement, Communication Scale; BL=Baseline; Wk24=Week 24; VP=Verbally-Prompted; NVP=Nonverbally Prompted; Spon.=Spontaneous. *This participant did not complete the week24 CGI. VM=Very Much Improved; M=Much Improved; Min=Minimally Improved; NC=No Change.

Supplemental Table S2.
Participant Scores on Relevant Predictor Variables

	Baseline Mullen Scales of Early Learning			Baseline Social Responsiveness Scale, 2nd Edition		Wk24 PRT Fidelity
Child	NVDQ	VDQ	FSIQ	RRB T-Score	SCI T-Score	
1	62.07	46.55	54.31	56.00	63.00	96.67
2	44.87	57.69	51.28	62.00	54.00	100.00
3	38.89	40.28	39.58	82.00	58.00	100.00
4	59.21	59.21	59.21	54.00	65.00	100.00
5	42.22	25.56	33.89	74.00	72.00	96.67
6	38.89	21.11	30.00	74.00	77.00	100.00
7	61.46	42.71	52.08	72.00	71.00	100.00
8	58.57	35.71	47.14	52.00	50.00	100.00
9	33.33	7.58	20.45	62.00	76.00	66.67
10	31.73	23.08	27.40	70.00	71.00	100.00
11	63.54	55.21	59.38	72.00	78.00	93.33
12	61.90	66.67	64.29	54.00	62.00	96.67
13	53.77	52.83	53.30	66.00	77.00	96.67
14	53.66	17.07	35.37	60.00	69.00	100.00
15	63.04	17.39	40.22	54.00	72.00	90.00
16	55.10	46.94	51.02	76.00	76.00	90.00
17	53.00	47.00	50.00	58.00	58.00	96.67
18	48.84	32.56	40.70	66.00	73.00	100.00
19	53.17	46.03	49.60	88.00	82.00	100.00
20	57.95	30.68	44.32	90.00	90.00	100.00
21	38.89	14.29	26.59	72.00	70.00	86.67
22	40.85	39.44	40.14	90.00	76.00	100.00
23	58.89	57.78	58.33	74.00	73.00	93.33

Note. NVDQ=Nonverbal Developmental Quotient; VDQ=Verbal Developmental Quotient; FSDQ=Full Scale Developmental Quotient. RRB = Restricted and repetitive behaviors. SCI=Social Communication and Interaction Index. Developmental Quotients calculated from baseline Mullen Scales of Early Learning scores.
